# Potential dose variability for small‐field plans delivered with Elekta Agility collimators

**DOI:** 10.1002/acm2.13349

**Published:** 2021-07-13

**Authors:** Joseph J. Foy, Poonam Yadav, Indra J. Das

**Affiliations:** ^1^ Department of Radiation Oncology Northwestern Memorial Hospital Northwestern University Feinberg School of Medicine Chicago Illinois 60611 USA

## CONFLICT OF INTEREST

There is no conflict of interest to disclose.

Dear Editor,

A recent publication by Lorenz and Paris^1^ “Identification of a potential source of error for 6 FFF beams delivered on an Agility^TM^ multileaf collimator” reported on discrepancy between calculated and measured doses to be 10% and 60% at isocenter for a 3.5‐mm and 1‐mm gap field size, respectively. The investigators attempted to determine the cause of the relatively high failure rates during their patient‐specific quality assurance (PSQA) when using flattening filter‐free (FFF) photon beams. The investigation involved simulating a number of sliding rectangular fields in Monaco^TM^ treatment planning system (TPS) and measuring the dose delivered by these plans with IBA’s Matrixx Evolution^TM^ detector. Additionally, they tested the range of VMAT and DCAT plans using ArcCheck^TM^. The authors concluded that the reduced passing rate for plans using FFF was due to the operation of the Agility MLC and its ability to violate leaf position tolerances.

We would like to highlight the statement from results section “In comparison, the TrueBeam^TM^ delivered all these fields according to plan and dosimetric agreement was well within specifications for all gap size.” The authors use this justification to focus on the Agility^TM^ MLC operation as the source of this error. We believe that this statement may be misleading to those using Elekta^TM^ linacs for radiotherapy treatments. At our institution, a Pinnacle^TM^ TPS is primarily used along with three Elekta^TM^ linacs (two with Agility^TM^ MLCs), one Truebeam^TM^, and Archeck^TM^ for PSQA. Both linacs underwent extensive MLC QA during commissioning per AAPM TG‐106^2^ with MLC positions abiding by a 1‐mm tolerance. Additionally, Kabat et al^3^ reported that Elekta Agility^TM^ MLC position accuracy can be satisfactorily tested within 0.1 mm tolerance using log files. The root mean error is dependent on the speed of the MLC (5 mm/s–35 mm/s) when varied linearly with MLC position errors ranging from 0.1 mm to 0.9 mm.

In the context of the publication by Lorenz and Paris,^1^ we retrospectively analyzed the PSQA results from 20 VMAT treatments planned with FFF and non‐FFF (referred to as FF) photon beams for prostate, brain, liver, lung, and head and neck tumors with varied complexity in modulation. Each treatment was planned on an Elekta Versa^TM^ (Agility^TM^ MLC) machine with Pinnacle^TM^ TPS and measured with ArcCheck^TM^. Planned and measured dose distributions were compared using gradient compensation, a 20% dose threshold, and 2.0%/2.0 mm dose difference and distance‐to‐agreement (DTA) criteria. The relative number of points passing these criteria were compared between plans created with and without the inclusion of the flattening filter. An analogous dataset of patients planned with FF and FFF photon beams on a Varian Truebeam^TM^ linac was obtained, and PSQA results were compared between two linacs.

In our study, the average PSQA passing rates on the Elekta Versa^TM^ were 90.6% and 94.1% for FFF and FF plans, respectively, and the passing rates on the Truebeam^TM^ were 91.1% and 96.2% for FFF and FF plans, respectively. Differences in passing rates between FF and FFF plans were statistically significant for both linacs (*p* = 0.002 and 0.004 for Elekta^TM^ and Truebeam^TM^, respectively); however, all plans were considered to be clinically acceptable.

While the differences in PSQA for FF and FFF photon beams require further investigation, one cannot necessarily conclude that differences in calculated and measured doses and the effect on PSQA passing rates are solely due to the operation of the Agility^TM^ MLC. Agreement in calculated and measured doses can vary with fluctuations in daily output, linac limitations, complexity of the measured plan, and a myriad of many other factors that have been extensively investigated.^4^, ^5^, ^6^, ^7^ Treatment plans using FFF beams such as stereotactic radiotherapy are generally highly modulated and typically use small fields compared to VMAT plans. When using ArcCheck^TM^, smaller fields also result in a reduced number of measurement points for comparison between calculated and measured dose distributions. These plans will subsequently reflect large variations in the passing rate when a small number of measurement points do not pass the 2.0%/2.0 mm criteria. Additionally, the Agility^TM^ MLC requires a gap between corresponding leaves of 5 mm when projected at isocenter. Therefore, the dose errors reported by Lorenz and Paris^1^ for field sizes smaller than 3.5 mm will not necessarily be found in most clinical plans. This limitation in leaf gap width may also be a source of discrepancy. If plans are created with gap sizes smaller than 5 mm, then the calculated dose distributions will differ from those that can be delivered with an Agility^TM^ MLC based on these limitations.

Finally, Lorenz and Paris^1^ found that the discrepancies in measured and calculated doses on the Elekta^TM^ linac were not found for similar plans on a Varian^TM^ linac. Our results indicated that these discrepancies persisted with either linac manufacturer with FFF plans having significantly lower passing rates than FF plans. This may indicate that their reported issues are not within the MLC design, but within the commissioning and beam modeling performed by the authors.

While the results obtained from the current study support the notion that additional investigations should analyze the effects of small fields, highly modulated fields, and the relationship between planned and measured dose grids on PSQA, they do not necessarily indicate that discrepancies in these measurements lie within the Agility^TM^ MLC itself. To fully understand the implications of differences in calculated and measured dose distributions, additional studies are required in the context of small‐field dosimetry and beam modeling, which TG‐155^8^ has addressed. While we respectfully suggest that the authors of the cited publication^1^ may have used unnecessarily alarming language that could otherwise undermine the confidence of the radiotherapy community, we are looking forward to the authors elaborating on their findings in future publications. Additional investigation may aid medical professionals in their greater understanding of the dependencies of PSQA on MLC and associated parameter operation, particularly for small fields.
